# MicroRNA-137 inhibits tumor growth and sensitizes chemosensitivity to paclitaxel and cisplatin in lung cancer

**DOI:** 10.18632/oncotarget.8011

**Published:** 2016-03-09

**Authors:** Hua Shen, Lin Wang, Xin Ge, Cheng-Fei Jiang, Zhu-Mei Shi, Dong-Mei Li, Wei-Tao Liu, Xiaobo Yu, Yong-Qian Shu

**Affiliations:** ^1^ Department of Oncology, The First Affiliated Hospital of Nanjing Medical University, Nanjing, Jiangsu, 210029, China; ^2^ Department of Thoracic Surgery, Shanghai General Hospital, Shanghai Jiao Tong University, Minhang, Shanghai, 200080, China; ^3^ State Key Laboratory of Reproductive Medicine, Nanjing Medical University, Nanjing, Jiangsu, 210029, China; ^4^ Department of Pathology, and Cancer Center, Nanjing Medical University, Nanjing, Jiangsu, 210029, China; ^5^ Department of Neurosurgery, The First Affiliated Hospital of Nanjing Medical University, Nanjing, Jiangsu, 210029, China; ^6^ Collaborative Innovation Center for Cancer Medicine, Jiangsu Key Lab of Cancer Biomarkers, Prevention and Treatment, Nanjing Medical University, Nanjing, Jiangsu, 210029, China

**Keywords:** miR-137, NUCKS1, paclitaxel, cisplatin, chemosensitivity

## Abstract

Chemotherapy resistance frequently drives tumour progression. However, the underlying molecular mechanisms are poorly characterized. In this study, we explored miR-137's role in the chemosensitivity of lung cancer. We found that the expression level of miR-137 is down-regulated in the human lung cancer tissues and the resistant cells strains: A549/paclitaxel(A549/PTX) and A549/cisplatin (A549/CDDP) when compared with lung cancer A549 cells. Moreover, we found that overe-expression of miR-137 inhibited cell proliferation, migration, cell survival and arrest the cell cycle in G1 phase in A549/PTX and A549/CDDP. Furthermore, Repression of miR-137 significantly promoted cell growth, migration, cell survival and cell cycle G1/S transition in A549 cells. We further demonstrated that the tumor suppressive role of miR-137 was mediated by negatively regulating Nuclear casein kinase and cyclin-dependent kinase substrate1(NUCKS1) protein expression. Importantly, miR-137 inhibits A549/PTX, A549/CDDP growth and angiogenesis *in vivo*. Our study is the first to identify the tumor suppressive role of over-expressed miR-137 in chemosensitivity. Identification of a novel miRNA-mediated pathway that regulates chemosensitivity in lung cancer will facilitate the development of novel therapeutic strategies in the future.

## INTRODUCTION

Lung cancer is the most frequent cause of cancer-related death both in China and in many other countries. Approximately 70%–80% of lung cancers are non-small cell lung cancer(NSCLC), including squamous cell carcinoma, adenocarcinoma, and large cell carcinoma [1, 2]. In NSCLC, the leading death cause is chemotherapy resistance and metastasis, yet the underlying mechanisms of them remain largely unclear [3–6]. At the molecular level, lung cancer arises from a series of genetic and epigenetic alterations that inactivate tumor suppressor genes and activate oncogenes. However, the basic mechanisms underlying lung cancer initiation and progression remain largely unknown. A greater understanding of the molecular mechanisms underlying carcinogenesis, progression and drug resistance in lung cancer would be helpful in improving diagnosis, therapy and prevention.

MicroRNAs (miRNA) are a class of small non-coding RNAs of 18–24 nucleotides that bind to partially complementary recognition sequences of mRNA, causing either degradation or inhibition of translation, thus effectively silencing their mRNA target [7]. Recently, miRNAs have been reported to be associated with chemoresistance of cancer, mainly through abnormal regulation of cell viability [8], cell apoptosis [9], cell cycle distribution [10], epithelial mesenchymal transition (EMT) [11] and cancer stem cell self-renewal [12, 13]. Among them, miR-137 has been demonstrated to function as a tumor suppressor, and loss of miR-137 expression has been reported in many cancer types, including myeloma, hepatocellular carcinoma, colorectal cancer, lung cancer and glioma [14–18]. Restoration of miR-137 expression has been shown to abrogate tumorigenesis. To date, some genes have been identified as miR-137 target genes, including Cdc42, Cdk6, Cox-2, paxillin, AKT2 and MCL-1 [14–18], which are involved in pathogenesis of cancers. However, the molecular mechanism of miR-137 repression in lung cancer has not been fully determined.

In the present study, we demonstrated that miR-137 was downregulated in 50 pairs human lung cancer specimens. Then, we will ask several important questions in this study: (1) what are the roles of miR-137 in lung cancer growth; (2) what are the roles of miR-137 in A549, A549/PTX and A549/CDDP cells; (3) what is the potential direct target of miR-137 that may be associated with cancer development; and (4) What role of miR-137 in chemoresistance of lung cancer *in vivo*. The answers of these questions would provide new insights into the molecular mechanism of lung cancer development as well as provide new therapeutic strategy for lung cancer treatment in the future.

## RESULTS

### Down-regulation of miR-137 expression in tumor tissues of human lung cancer patients

**Figure 1:**
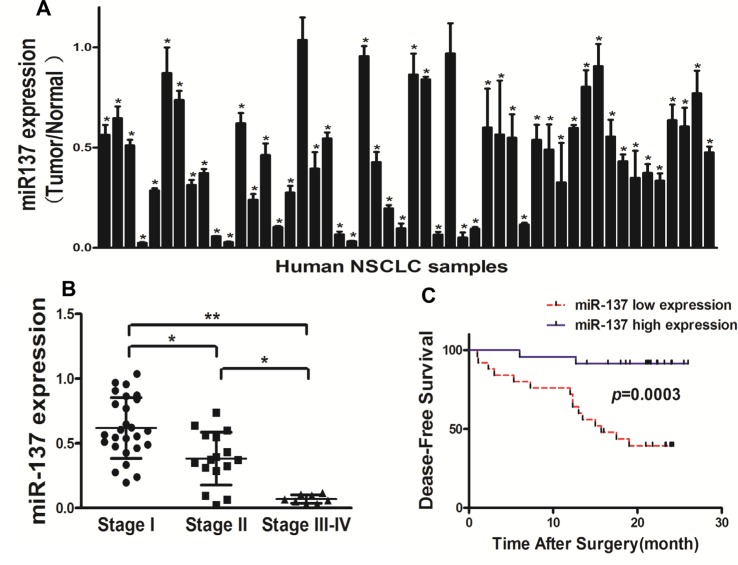
Down-regulation of miR-137 expression in tumor tissues of human lung cancer patients (**A**) Expression levels of miR-137 in 50 pairs of lung cancer tumor and adjacent normal specimens were analyzed by stem-loop qRT-PCR, and normalized to the levels of U6. The fold changes were obtained by the ratio of miR-137 in cancer tissues to that in the adjacent normal tissues. *P < 0.05 comparing miR-137 expression in tumor tissues with adjacent normal tissues. (**B**) Relative expression levels of miR-137 in different stages of cancer tissues. (**C**) Kaplan-Meier curves depicting Disease-free survival according to expression of miR-137. The cutoff for the definition of subgroups (high and low) of miR-137 expression level was the 50th percentile value.

We tested the expression levels of miR-137 in 50 pairs of non-small cell lung cancer(NSCLC) tumor specimens and adjacent normal tissues, and found that miR-137 expression levels in tumor tissues were significantly lower than those controls (Figure 1A). The expression levels of miR-137 were significantly lower in WHO stage III-IV NSCLC tissues than those in stage I and stage II, indicating that miR-137 expression was greatly down-regulated in late stages of lung cancer cancer tissues (Figure 1B). To further evaluate the correlation between miR-137 expression levels and prognosis in NSCLC patients, we used Kaplan-Meier survival analysis and log-rank test to assess the normalized miR-137 expression levels (tumor/normal) and Disease free survival (DFS). The results showed that patients with low miR-137 expression levels had a shorter disease free survival (DFS) compared with patients with high miR-137 expression (Figure 1C). Taken together, the low expression levels of miR-137 in lung cancer patients could be used as a potential new biomarker which could predict poor prognosis for NSCLC.

### A549/PTX and A549/CDDP show stronger activity of proliferation, migration and cell cycle progression, lower apoptosis activity when compared with A549 cells

**Figure 2:**
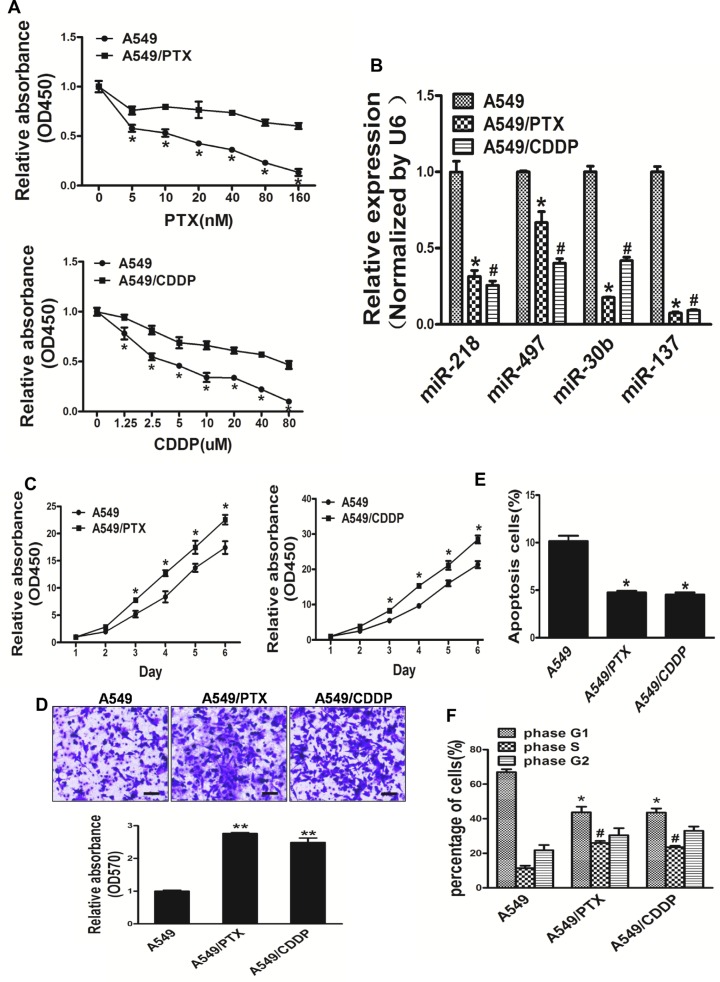
A549/PTX and A549/CDDP show stronger activity of proliferation, migration and cell cycle progression, lower apoptosis activity when compared with A549 cells (**A**) Compared with A549 cells, A549/PTX and A549/CDDP cells displayed less sensitive to paclitaxel and cisplatin, respectively. (**B**) The levels of 4 miRNA expression in lung cancer cells A549 and resistant cells strains: A549/PTX, A549/CDDP. (**C**) The CCK8 assays of A549, A549/PTX and A549/CDDP cells were determined in various time points, respectively. (**D**) Transwell migration assays was conducted in respective cells. (**E**) Apoptosis Assay were conducted in A549, A549/PTX and A549/CDDP cells. (**F**) Cell cycle analysis were conducted in A549, A549/PTX and A549/CDDP cells. Data represent mean ± SD. of 3 replicates. * indicated *P* < 0.05; **indicated *P* < 0.01.

Paclitaxel and cisplatin-based chemotherapy have been the cornerstone of treating advanced lung cancer. In order to mimic the pathophysiological impact of long-time exposure to paclitaxel and cisplatin, which are the firstline medicines in the treatment of lung cancer, we established A549/PTX and A549/CDDP cell lines *in vitro* model by transforming human lung cancer A549 cells via exposure to indicated lower concentration paclitaxel and cisplatin for 24 weeks. Comparing the A549 cell line the two established resistant cells separately showed drug resistance to PTX and CDDP (Figure 2A). We tested the expression levels of miR-218, miR-497, miR-30b and miR-137 in A549, A549/CDDP and A549/PTX cell lines. Interestingly, the expression levels of miR-137 in lung cancer A549 cells were higher than resistant cells strains: A549/PTX and A549/CDDP (Figure 2B). A549/PTX and A549/CDDP cells showed the characteristics of resistant cells such as increased activity of cell proliferation, migration, cell cycle progression and lower apoptosis activity (Figure 2C–2F). In our study, we found that A549/PTX and A549/CDDP show stronger activity of proliferation, migration and cell cycle progression, lower apoptosis activity when compared with A549 cells.

### Repression of miR-137 in A549 cells signifcantly promoted cell growth, migration, cell survival, cell cycle G1/S transition and rendered resistance to PTX and CDDP

**Figure 3:**
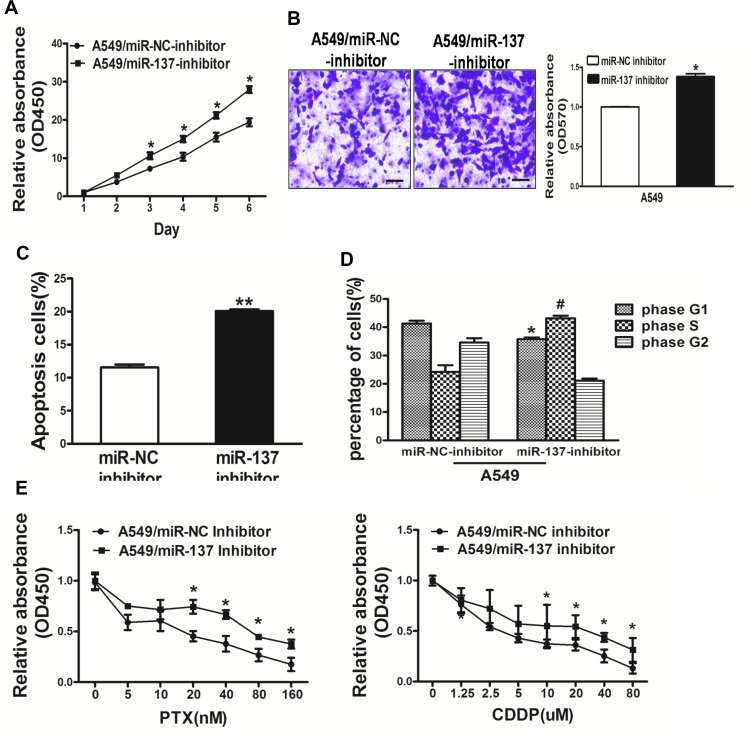
Repression of miR-137 in A549 cells signifcantly promoted cell growth, migration, cell survival and cell cycle G1/S transition and rendered resistance to PTX and CDDP (**A**) The CCK8 assay of A549 cells were determined after transduction with the miR-137 or miR-NC inhibitors, respectively. (**B**) Transwell migration assays were conducted in respective cells. (**C**) Apoptosis Assay were conducted in respective cells. (**D**) Cell cycle analysis were conducted in respective cells. (**E**) PTX and CDDP sensitivity in A549/miR-NC inhibitor, A549/miR-137 inhibitor cell lines tested by CCK-8 assay. Data represent mean ± SD. of 3 replicates. *indicated *P* < 0.05; **indicated *P* < 0.01, ^#^ indicated *P* < 0.05.

To study the role of miR-137 in lung cancer carcinogenesis, A549 cells transfected with miR-137-inhibitor were used to analyze cell growth. The results showed that the activity of cell growth in A549 cells were enhanced when inhibition of miR-137 expression compared with A549 cells expressing miR-NC (Figure 3A).

Since migration is key characteristics of malignant tumor, we next investigated the effects of miR-137 on cell migration. miR-137-inhibitor dramatically inhibited the normally strong migration capacity of lung cancer cells (Figure 3B). Moreover, inhibition of miR-137 expression promoted cell survival by Apoptosis assay and cell cycle G1/S transition by Cell Cycle analysis, respectively (Figure 3C, 3D). We further found that inhibition of miR-137 could render resistance to PTX and CDDP in A549 cell lines (Figure 3E). Thus, our results suggest that repression of miR-137 in A549 cells signifcantly promoted cell growth, migration, cell survival, cell cycle G1/S transition and chemo-resistance in A549 cell lines.

### Overexpression of miR-137 in A549/PTX and A549/CDDP cells inhibited cell proliferation, migration, induced cell apoptosis, arrest the cell cycle in G1 phase and reversed drug resistance to PTX and CDDP in A549/PTX and A549/CDDP cell lines respectively

**Figure 4:**
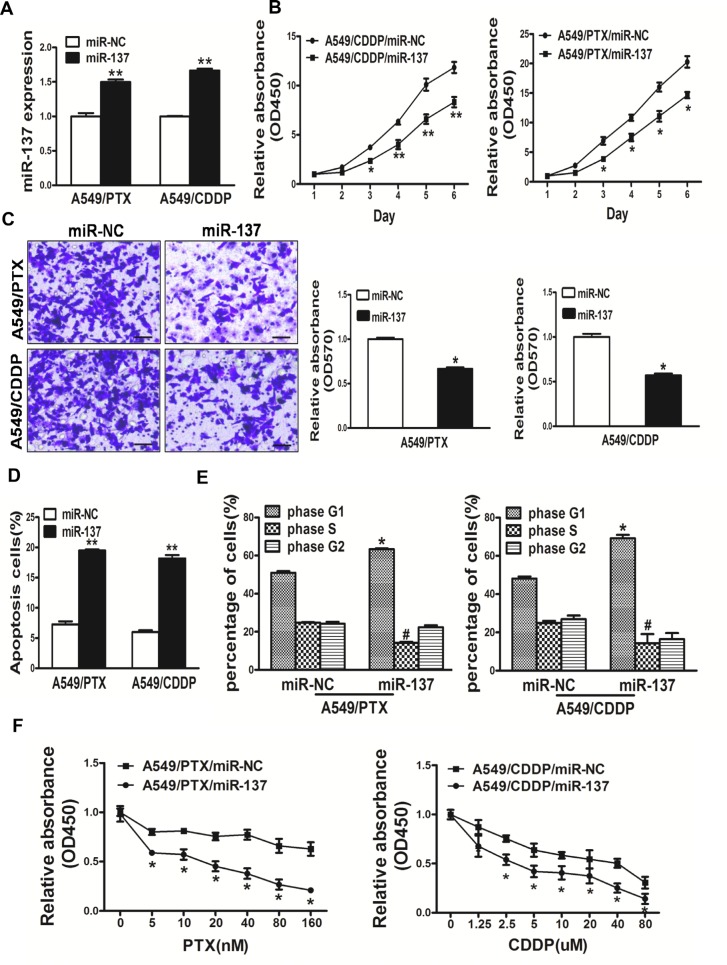
Overexpression of miR-137 in A549/PTX and A549/CDDP cells inhibited cell proliferation, migration, induced cell apoptosis and arrest the cell cycle in G1 phase and reversed drug resistance to PTX and CDDP in A549/PTX and A549/CDDP cell lines respectively (**A**) Real-time PCR analysis to quantify the expression levels of miR-137 and in A549/PTX and A549/CDDP cells. (**B**) The CCK8 assay of cells were determined after transduction with the miR-137 or miR-NC, respectively. (**C**) Transwell migration assays were conducted in respective cells. (**D**) Apoptosis Assay were conducted in respective cells. (**E**) Cell cycle analysis were conducted in respective cells. (**F**) PTX and CDDP sensitivity in A549/CDDP/miR-NC, A549/CDDP/miR-137, A549/PTX/miR-NC, A549/PTX/miR-137 cell lines tested by CCK-8 assay. Data represent mean ± SD. of 3 replicates. *indicated *P* < 0.05; **indicated *P* < 0.01, ^#^ indicated *P* < 0.05.

The expression levels of miR-137 in resistant cells strains of lung cancer A549/PTX and A549/CDDP were lower than A549 cells. In this study, miR-137 overexpressing A549/PTX and A549/CDDP cells were used to analyze cell growth (Figure 4A). The results showed that cell growth were attenuated in miR-137-overexpressing lung cancer cells compared with lung cancer cells expressing miR-NC (Figure 4B). We next investigated the effects of miR-137 on cell migration. Restoration of miR-137 dramatically inhibited the normally strong migration capacity of lung cancer cells (Figure 4C). What's more, overexpression of miR-137 promoted cell apotosis by Apoptosis assay and arrest the cell cycle in G1 phase by Cell Cycle analysis, respectively (Figure 4D, 4E). We further found that overexpression of miR-137 could reverse drug resistance to PTX and CDDP in A549/PTX and A549/CDDP cell lines respectively. Thus, our results suggest that overexpression of miR-137 in A549/PTX and A549/CDDP cells inhibited cell proliferation, migration, induced cell apotosis, arrest the cell cycle in G1 phase and reversed drug resistance to to PTX and CDDP in A549/PTX and A549/CDDP cell lines respectively.

### NUCKS1 is a direct target of miR-137, and is elevated in human lung cancer tissues, which is inversely correlated with miR-137 expression levels

**Figure 5:**
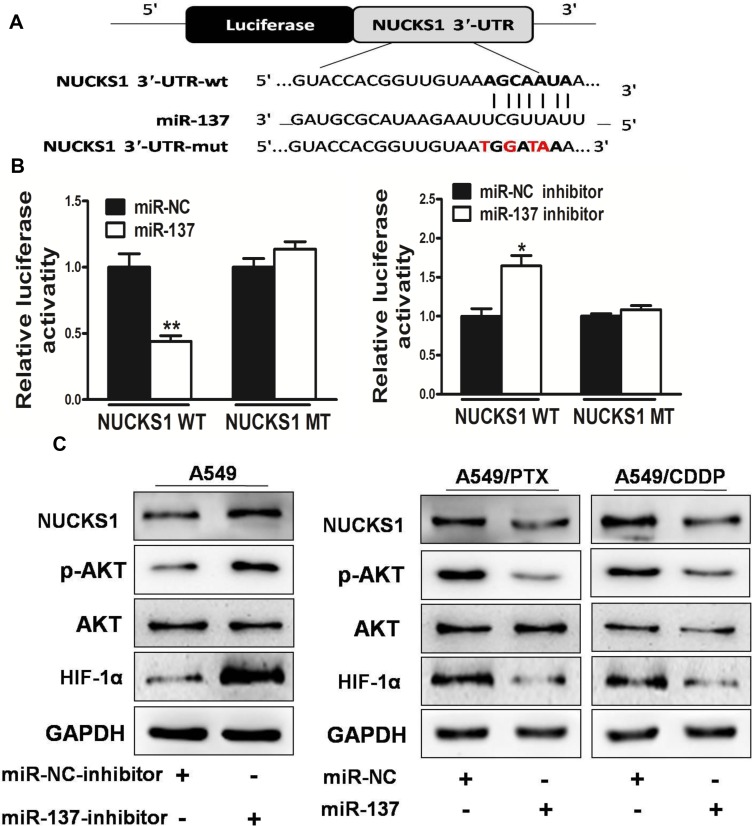

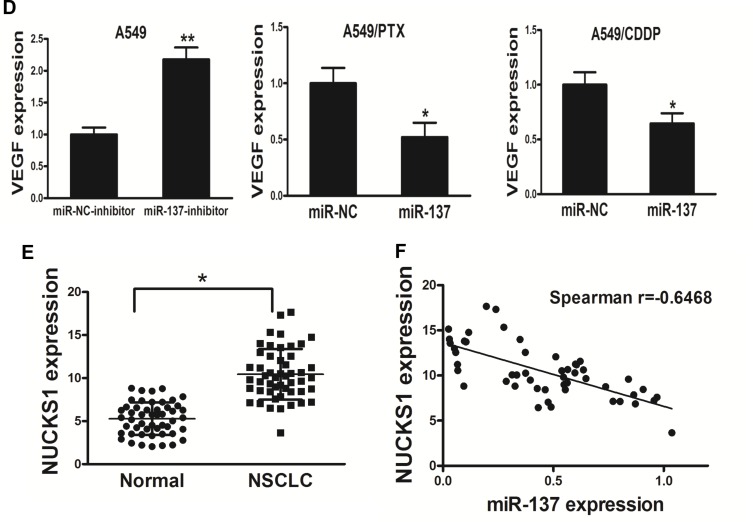
NUCKS1 is a direct target of miR-137, and is elevated in human lung cancer tissues, which is inversely correlated with miR-137 expression levels (**A**) Sequence of the miR-137-binding site within the human NUCKS1 3′-UTR and a schematic diagram of the reporter construct showing the entire NUCKS1 3′-UTR sequence and the mutated NUCKS1 3′-UTR sequence. The mutated nucleotides of the NUCKS1 3′-UTR are labeled in red. (**B**) Luciferase assay on A549 cells, which were co-transfected with miR-NC or miR-137 and a luciferase reporter containing the full length of NUCKS1 3′-UTR (WT) or a mutant (Mut) in which four nucleotides of the miR-137-binding site were mutated. Luciferase activities were measured 24 hours post transfection. MiR-137 markedly suppressed luciferase activity in NUCKS1 3′-UTR (WT) reporter constructs. In addition, miR-137 inhibitor induced luciferase activity in NUCKS1 3′-UTR (WT) reporter constructs. The data are means ± SEM. for separate transfections (*n* = 4). (**C**) The immunoblotting showed that expression levels of NUCKS1 were decreased in A549/PTX and A549/CDDP cells with miR-137 overexpression, but increased in A549 cells with miR-137 inhibitor. The expression levels of phosphorylated AKT (p-AKT) and HIF-1α were decreased in A549/PTX and A549/CDDP cells with miR-137 overexpression, but increased in A549 cells with miR-137 inhibitor, while AKT and protein levels were not changed. (**D**) The expression levels of VEGF were measured by qRT-PCR and normalized to GAPDH. The expression levels of VEGF were decreased in A549/PTX and A549/CDDP cells with miR-137 overexpression, but increased in A549 cells with miR-137 inhibitor. (**E**) The expression levels of NUCKS1 in normal tissues and human lung cancer specimens were determined by Western blotting analysis, the density signals were quantified using ImageJ software, and fold changes were obtained by the ratios of NUCKS1 to GAPDH levels. (**F**) Spearman′s correlation analysis was used to determine the correlations between the expression levels of NUCKS1 and miR-137 in human lung cancer specimens. Data represent mean ± SD. of 3 replicates. *indicated *P* < 0.05; **indicated *P* < 0.01.

To fully understand the mechanisms of miR-137 in lung cancer, TargetScan search program was used to predict targets of miR-137, which NUCKS1 has been thought to be putative target of miR-137 (Figure 5A). A549 cells were cotransfected with the wild (WT) or mutated (Mut) NUCKS1 luciferase reporter vector together with miR-137 or miR-NC for 24 h, and luciferase activities in those cells were measured. As shown in Figure 5B, luciferase activities were significantly reduced in those cells transfected with the wild sequence and miR-137, and significantly induced in those cells transfected with the wild sequence and miR-137 inhibitor, but not in the cells with the mutant sequence. Then, western blotting analysis was conducted to measure the levels of NUCKS1 protein, we found that the expression of NUCKS1 protein was downregulated in miR-137 treated cells, but increased in cells transfected with miR-137 inhibitor (Figure 5C). These results suggest that miR-137 directly targets NUCKS1 by binding its seed region to their 3′-UTRs in lung cancer cells. The PI3K/AKT pathways play important role in carcinogensis and several downstream factors such as hypoxia-inducible factor-1α (HIF-1α) and VEGF have been linked to the PI3K/AKT pathways. Cellular levels of p-AKT were significantly decreased in lung cancer cells stably expressing miR-137 compared with miR-NC, while no statistically significant reduction of AKT was detected (Figure 5C). Here, we observed that the expression levels of p-AKT, HIF-1α and VEGF in miR-137-expressing lung cancer cells were both downregulated, but elevated in miR-137-inhibitor expressing lung cancer cells (Figure 5C, 5D). These results suggest that miR-137 inhibits PI3K/AKT pathways via targeting NUCKS1.

Furthermore, we measured the levels of NUCKS1 proteins in human lung cancer specimens and normal tissues. The results showed that the average expression levels of NUCKS1 were significantly higher in tumor tissues than those in the normal tissues (Figure 5E). Then, we determine the correlation between NUCKS1 levels and miR-137 expression levels in the same human lung cancer specimens. As shown in Figure 5F, Spearman's rank correlation analysis showed that the expression levels of NUCKS1 and miR-137 in 50 human lung cancer specimens were inversely correlated (Spearman's correlation *r* = −0.6468).

### MiR-137 enhances the chemosensitivity of paclitaxel and cisplatin *in vivo*

**Figure 6:**
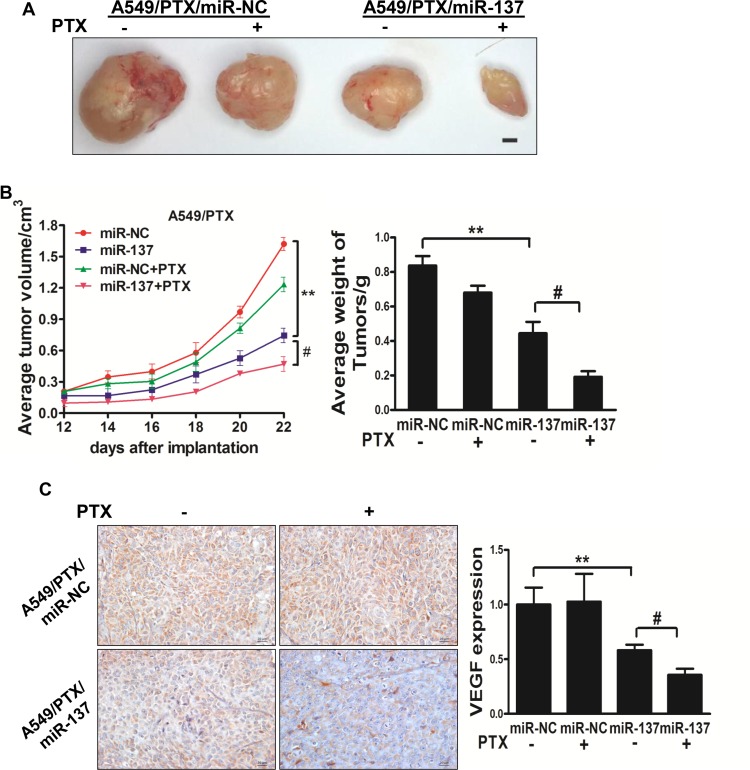

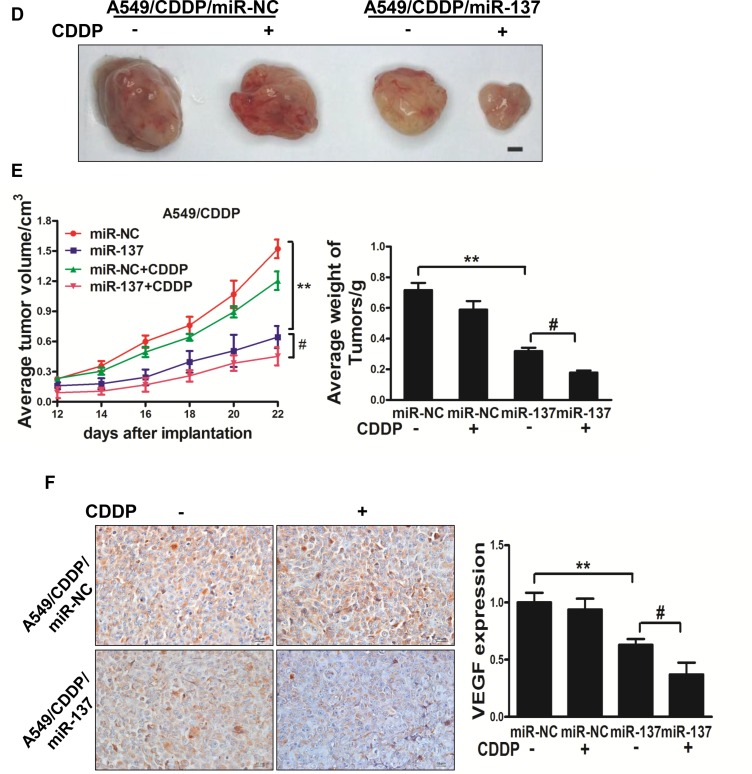
MiR-137 enhances the chemosensitivity of paclitaxel and cisplatin *in vivo* (**A, B**) Effect of miR-137 on the growth of A549/PTX cells inoculated into nude mice. Male BALB/c nude mice were subcutaneously injected with 5 × 10^6^ cells infected with lentiviruses harboring miR-NC or miR-137. Tumor volume was monitored over time as indicated, then PTX was added by peritoneal injection. The tumor was excised and weighed after 24 days. MiR-137 causes a decrease in tumor volume and weight when compared with miR-NC, and miR-137 plus PTX causes a decrease in tumor volume and weight when compared with miR-137. Bar = 1 mm. (**C**) The expression levels of VEGF were analyzed in A549/PTX tumor tissues by immunohistochemistry with representative images showed. The expression levels of VEGF was quantified by qRT-PCR. Magnification, ×200, Scale bar, 20 μm. Data were presented by mean ± SD. **indicated *P* < 0.01, ^#^indicated *P* < 0.05. (**D, E**) Effect of miR-137 on the growth of A549/CDDP cells inoculated into nude mice. Male BALB/c nude mice were subcutaneously injected with 5 × 10^6^ cells infected with lentiviruses harboring miR-NC or miR-137. Tumor volume was monitored over time as indicated, then CDDP was added by peritoneal injection. The tumor was excised and weighed after 24 days. MiR-137 causes a decrease in tumor volume and weight when compared with miR-NC, and miR-137 plus CDDP causes a decrease in tumor volume and weight when compared with miR-137. Bar = 1 mm. (**F**) The expression levels of VEGF were analyzed in A549/CDDP tumor tissues by immunohistochemistry with representative images showed. The expression levels of VEGF was quantified by qRT-PCR. Magnification, ×200, Scale bar, 20 μm. Data were presented by mean ± SD. **indicated *P* < 0.01, ^#^indicated *P* < 0.05.

To further investigate the role of miR-137 in tumor growth *in vivo*, ectopic transplantation model of human lung cancer in nude mice was employed. Stable cell lines, A549/PTX/miR-NC, A549/PTX/miR-137, A549/CDDP/miR-NC and A549/CDDP/miR-137 were collected and subcutaneously injected into both posterior blanks of male BALB/c nude mice, respectively. Tumor volumes were monitored every 2 days during the tumor inoculation period, then PTX or CDDP was added by peritoneal injection. The tumor was excised and weighed after 24 days. MiR-137 causes a decrease in tumor volume and weight when compared with miR-NC, and miR-137 plus PTX or CDDP causes a decrease in tumor volume and weight when compared with miR-137 (Figure 5A, 5B, 5D, 5E). To reveal the molecular mechanisms of miR-137 in tumor growth, total RNAs were extracted to perform qPCR analysis and found that the expression levels of VEGF, a key angiogenic regulator in both tumorigenesis and angiogenesis, were differentially regulated in these 2 groups of tumors (Figure 5C, 5F). Moreover, the tumors from A549/PTX/miR-137 plus PTX and A549/CDDP/miR-137 plus CDDP showed an decreased VEGF positive staining through a standard immunohistochemistry staining (Figure 5C, 5F). These results suggested that miR-137 enhances lung cancer paclitaxel and cisplatin sensitivity in nude mice Figure 6.

## DISCUSSION

In both the United States and Canada, lung cancer is by far the leading cause of cancer death [19, 20]. Lung carcinogenesis is a multistep process, which results from activation of oncogenes and inactivation of tumor suppressor genes [21]. The 5-year relative survival rate for lung cancer is only 17%, because in a high proportion of patients the disease is already metastatic at diagnosis [19]. Metastatic lung cancer is generally incurable because it will either have intrinsic resistance to chemotherapy or else will develop acquired resistance after an initial response [22].

MicroRNAs (miRNAs) have been documented to function both as tumor suppressor genes and oncogenes, regulating many cellular events. Recent studies have been reported that miR-137 plays a potential role as a tumor suppressor in many kinds of cancers. In this study, we found that the expression of miR-137 is downregulated in lung cancer samples compared with adjacent normal tissues. The expression level of miR-137 is downregulated in the lung cancer resistant cells strains: A549/PTX and A549/CDDP when compared with lung cancer A549 cells. Moreover, we found that overexpression of miR-137 inhibited cell proliferation, migration, induced apotosis and arrested the cell cycle in G1 phase in A549/PTX and A549/CDDP; repression of miR-137 signifcantly promoted cell growth, migration, cell survival and cell cycle G1/S transition in A549 cells. Our study is the first to identify the tumor suppressive role of overexpressed miR-137 in chemosensitivity. Identification of a novel miRNA-mediated pathway that regulates chemosensitivity in lung cancer will facilitate the development of novel therapeutic strategies in the future.

NUCKS1 is a nuclear, DNA-binding and highly phosphorylated protein. It has been shown that NUCKS is a substrate for CK2, Cdk1 and DNA-activated Kinase *in vitro* and *in vivo* [23–26]. NUCKS1 appears to be important for cell cycle progression [27] and is involved in facilitating and maintaining the transcription activity of some genes during rapid proliferation [28]. Increased NUCKS1 expression has been reported in several cancers [29–33], including ovarian cancer [28]. However, the status of NUCKS1 expression in lung cancer remains unknown. In our study, NUCKS1 oncogene has been experimentally validated as the novel target of miR-137 not only *in vitro*, but also *in vivo*.

Paclitaxel and cisplatin-based chemotherapy have been the cornerstone of treating advanced lung cancer for over a half century [34, 35]. Recent studies have focused on the involvement of microRNA during the acquisition of chemoresistance in cancer. MiRNAs are differentially expressed in chemosensitive and chemoresistant cells [36, 37]. Recent study showed that ectopic miR-34a sensitized the colorectal cancer cells to 5-fluorouracil [38]. MiR-124 enhances chemosensitivity by targeting R-RAS and N-RAS [39]. In this study, we found that a miR-137 restoration approach may offer a new modulation strategy to overcome chemoresistance to paclitaxel and cisplatin in lung cancer.

In summary, we have identified a link between miR-137, NUCKS1 and chemoresistance that is a novel constituent of lung cancer tumorigenesis. Over the past few years, it has been shown that the miR-137 was downregulated in different tumors such as hepatocellular carcinoma, colorectal cancer, lung cancer and glioma. We speculate that these tissue-specific miRNAs may contribute to the cognate abnormality *via* similar pathways. Now despite having a better understanding of the molecular events that govern the lung cancer than ever before, it remains a clinical challenge in the treatment of lung cancer. As paclitaxel and cisplatin-based chemotherapy is still the main treatment option for advanced metastatic lung cancer, it is important that a miR-137 restoration approach may offer a new modulation strategy to overcome chemoresistance.

## MATERIALS AND METHODS

### Clinical specimens

Paired human lung cancer specimens and matched normal adjacent tissue samples were collected from patients undergoing a surgical procedure in the First Affiliated Hospital of Nanjing Medical University, Nanjing, China, with the informed consent of the patients. Parts of tissue samples were immediately snap-frozen in liquid nitrogen, and parts were fixed in formalin for histological examination. All samples were histologically classified by clinical pathologist. The experiment protocols have been approved by the ethics committees of Nanjing Medical University, Nanjing, China.

### Cell culture and reagents

Human lung cancer cell lines, A549, A549/PTX and A549/CDDP were maintained in RPMI1640 medium supplemented with 10% fetal bovine serum and antibiotics (100 units/ml penicillin and 100 mg/ml streptomycin). HEK293T cells was cultured in DMEM medium supplemented with 10% fetal bovine serum, 100 units/ml of penicillin and 100 mg/ml of streptomycin. Cells were incubated at 37°C in a humidified atmosphere of 5% CO_2_ in air. Antibodies against p-AKT (Ser473) and AKT were purchased from Cell Signaling Technology (Danvers, MA, USA). Antibodies against HIF-1α and GAPDH were from Bioworld Technology (Atlanta, Georgia 30305, USA), and antibodies against NUCKS1 were from Sigma-Aldrich (St. Louis, MO, USA), respectively.

### Lentivirus packaging and stable cell lines

The lentiviral packaging kit was purchased from Open Biosystems (Huntsville, AL, USA). Lentivirus carrying hsa-miR-137 or hsa-miR-negative control (miR-NC) was packaged following the manufacturer's manual. Lentivirus were packaged in HEK293T cells and collected from the medium supernatant. Stable cell lines were established by infecting lentivirus into A549/PTX and A549/CDDP cells and selected by puromycin.

### RNA extraction, reverse transcription PCR and quantitative real time-PCR

RNA was isolated from harvested cells or human tissues with Trizol reagent according to the manufacturer's instruction (Invitrogen, CA, USA). To measure expression levels of miR-137, stem-loop specific primer method was used as follows: forward primer: GCTCCTCAGGTCGAACCTATTG; Reverse primer: CCGACGCTATTGCTTAAGAATACG. Expression of U6 was used as an endogenous control. To determine the mRNA levels of VEGF, total RNAs were reversely transcribed by oligodT primer using PrimeScript RT Reagent Kit (Vazyme, Nanjing, China). Housekeeping gene GAPDH was used as internal control, primers were used as described before [40]. The cDNAs were amplified by qRT-PCR using AceQ SYBR Master Mix (Vazyme, Nanjing, China) on a 7900HT system, and fold changes were calculated by relative quantification (2^−ΔΔCt^).

### Immunoblotting

Cells were washed with ice-cold PBS buffer, scraped from the dishes, and centrifuged at 12,000 rpm, 4°C for 15 min. Cell lysates were prepared using RIPA buffer supplemented with protease inhibitors (100 mM Tris, pH 7.4, 150 mMNaCl, 5 mM EDTA, 1% Triton X-100, 1% deoxycholate acid, 0.1% SDS, 2 mM phenylmethylsulfonyl fluoride, 1 mM sodium orthovanadate, 2 mM DTT, 2 mMleupeptin, 2 mMpepstatin). The supernatants were collected and protein concentration was determined using BCA assay (Beyotime Institute of Biotechnology, Jiangsu, China). Tumor tissues from human and nude mice were grinded into powder in liquid nitrogen with RIPA buffer, and the total tissue proteins were extracted as described above. Aliquots of protein lysates were fractionated by SDS-PAGE, transferred to a PVDF membrane (Roche, Switzerland), and subjected to immunoblotting analysis according to the manufacturer's instruction. ECL Detection System (Thermo Scientific, Rockford, IL, USA) was used for signal detection.

### Luciferase reporter assay

The 3′-UTR of NUCKS1 were synthesized and annealed, then inserted into the SacI and HindIII sites of pMIR-reporter luciferase vector (Ambion) at downstream of the stop codon of the gene for luciferase. For its mutagenesis, the sequences complementary to the binding site of miR-137 in the 3′-UTR (NUCKS1: AGCAATAA) was replaced by TGGATAAA. These constructs were validated by sequencing. A549 cells were seeded into a 24-well plate for luciferase assay. After cultured overnight, cells were cotransfected with the wild-type or mutated plasmid, pRL-TK plasmid, and equal amounts of miR-137 mimics, miR-NC, and miR-137 inhibitors or miR-NC inhibitors, respectively. Luciferase assays were performed 24 h after transfection using the Dual Luciferase Reporter Assay System (Promega, WI, USA).

### Cell proliferation

To determine the effects of miR-137 on growth of lung cancer cells, cells were seeded in 96-well plates at confluence of 2000 cells per well. The absorptions of the cells were measured using a CCK8 kit (Dojindo Laboratories, Kumamoto, Japan) according to the manufacturer's instruction at different indicated time points. Data were from three separate experiments with four replications each time.

### Cell migration assay

Migration assay was determined using 24-well BD Matrigel migration chambers (BD Biosciences, Cowley, UK) in accordance with the manufacturer's instructions. 5 × 10^4^ cells were seeded per well in the upper well of the migration chamber in RPMI1640 without serum, the lower chamber well contained RPMI1640 supplemented with 10% FBS to stimulate cell migration. After incubation for 24 h, noninvading cells were removed from the top well with a cotton swab while the bottom cells were fixed with 3% paraformaldehyde, stained with 0.1% crystal violet, and photographed in three independent fields for each well. They were finally extracted with 33% acetic acid and detected quantitatively using a standard microplate reader (at 570 nm). Three independent experiments were conducted in triplicate.

### *In vitro* chemosensitivity assay

Cancer cells were seeded at a density of 4,000 cells per well in a 96-well plate overnight. Freshly prepared Paclitaxel (Sigma-Aldrich, St. Louis, MO, USA) was added with the final concentration ranging from 5 to 160 nM. Freshly prepared CDDP (Sigma-Aldrich, St. Louis, MO, USA) was added with the final concentration ranging from 1.25 to 80 μM. 48 h later, cell viability was assayed by CCK8 kit.

### Apoptosis assay

Apoptosis were measured by flow cytometry as described before [41]. For AnnexinV staining, 5 μL phycoerythrin-Annexin V, 5 μL propidium iodide (BD Pharmingen) and 400 μL 1 × binding buffer were added to the samples, which were incubated for 15 min at room temperature in the dark. Then the samples were analyzed by flow cytometry (FACSCanto II, BD Biosciences) within 1 h. The data were analyzed using FlowJo software. Three experiments were performed in triplicate.

### Tumorigenesis in nude mice

Male BALB/c nude mice (6-weeks-old) were purchased from Shanghai Laboratory Animal Center (Chinese Academy of Sciences, Shanghai, China) and maintained in special pathogen-free (SPF) condition for one week. Animal handling and experimental procedures were in accordance with the Guide for the Care and Use of Laboratory Animals, and approved by the Animal Experimental Ethics Committee of Nanjing Medical University. A549/PTX and A549/CDDP cells stably expressing miR-137 or miR-NC were injected subcutaneously into both flanks of nude mice (5×10^6^ cells in 100 μl). Tumor sizes were measured using vernier caliper every two days when the tumors were apparently seen and tumor volume was calculated according to the formula: volume = 0.5 × Length × Width^2^. Ten days after implantation, paclitaxel (10 nM) or cisplatin (5 μM) was intraperitoneal injectd in indicated mice. 24 days after implantation, mice were sacrificed and tumors were dissected. Tumors were formalin-fixed, paraffin-embedded, and sectioned at 5 μm for VEGF (Santa Cruz, CA, USA) immunohistochemical staining under the standard procedure as described before [42].

### Statistical analysis

All experiments were performed three times and data were analyzed with GraphPad Prism 5(La Jolla, CA, USA). The correlation between miR-137 expression and NUCKS1 levels in human lung cancer tissues were analyzed using Spearman's rank test. Kaplan-Meier survival analysis and log-rank test were used to assess the normalized miR-137 expression levels (tumor/normal) and Disease free survival (DFS). Statistical evaluation for data analysis was determined by *t*-test. The differences were considered to be statistically significant at *P* < 0.05.
